# Extraordinarily Slow, Non‐Metastatic Progression of Primary Mediastinal Small Cell Carcinoma

**DOI:** 10.1002/rcr2.70577

**Published:** 2026-05-11

**Authors:** Seo In Ahn, Jae Seok Jeong, Myeong Ja Chung, Kum Ju Chae, Yong Chul Lee

**Affiliations:** ^1^ The Catholic University of Korea College of Medicine Seocho‐gu South Korea; ^2^ Department of Internal Medicine, Research Center for Pulmonary Disorders Jeonbuk National University Medical School Jeonju South Korea; ^3^ Research Institute of Clinical Medicine of Jeonbuk National University‐Biomedical Research Institute of Jeonbuk National University Hospital Jeonju South Korea; ^4^ Respiratory Drug Development Research Institute Jeonbuk National University Medical School Jeonju South Korea; ^5^ Laboratory of Respiratory Immunology and Infectious Diseases, Korea Zoonosis Research Institute Jeonbuk National University Iksan South Korea; ^6^ Department of Pathology Jeonbuk National University Medical School Jeonju South Korea; ^7^ Department of Radiology Jeonbuk National University Medical School Jeonju South Korea

**Keywords:** lymphadenopathy, mediastinal mass, small cell carcinoma

## Abstract

We report a rare untreated mediastinal small cell carcinoma (MSCC) showing unusually slow, non‐metastatic progression over long‐term CT follow‐up. MSCC should be considered in mediastinal mass differentials. Distinguishing malignancy from benign lymphadenopathy in patients with lung disease is essential to avoid diagnostic delays.

## Graphical abstract

This case demonstrates the extremely slow and non‐metastatic progression of untreated mediastinal small cell carcinoma over long‐term follow‐up. This highlights the importance of distinguishing between rare malignant tumours and benign lymphadenopathy to enable timely treatment.

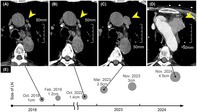

A 79‐year‐old male was diagnosed with a mediastinal malignant mass abutting the aortic arch. The patient had a 49‐pack‐year history of smoking and was receiving treatment for chronic obstructive pulmonary disease and recurrent pneumonia. Serial computed tomography (CT) scans over several years demonstrated progressive enlargement and invasion of para‐aortic lymph node (Figure 1A–D). A diagnostic evaluation for malignancy was recommended in 2023 but was declined by the patient.

**FIGURE 1:**
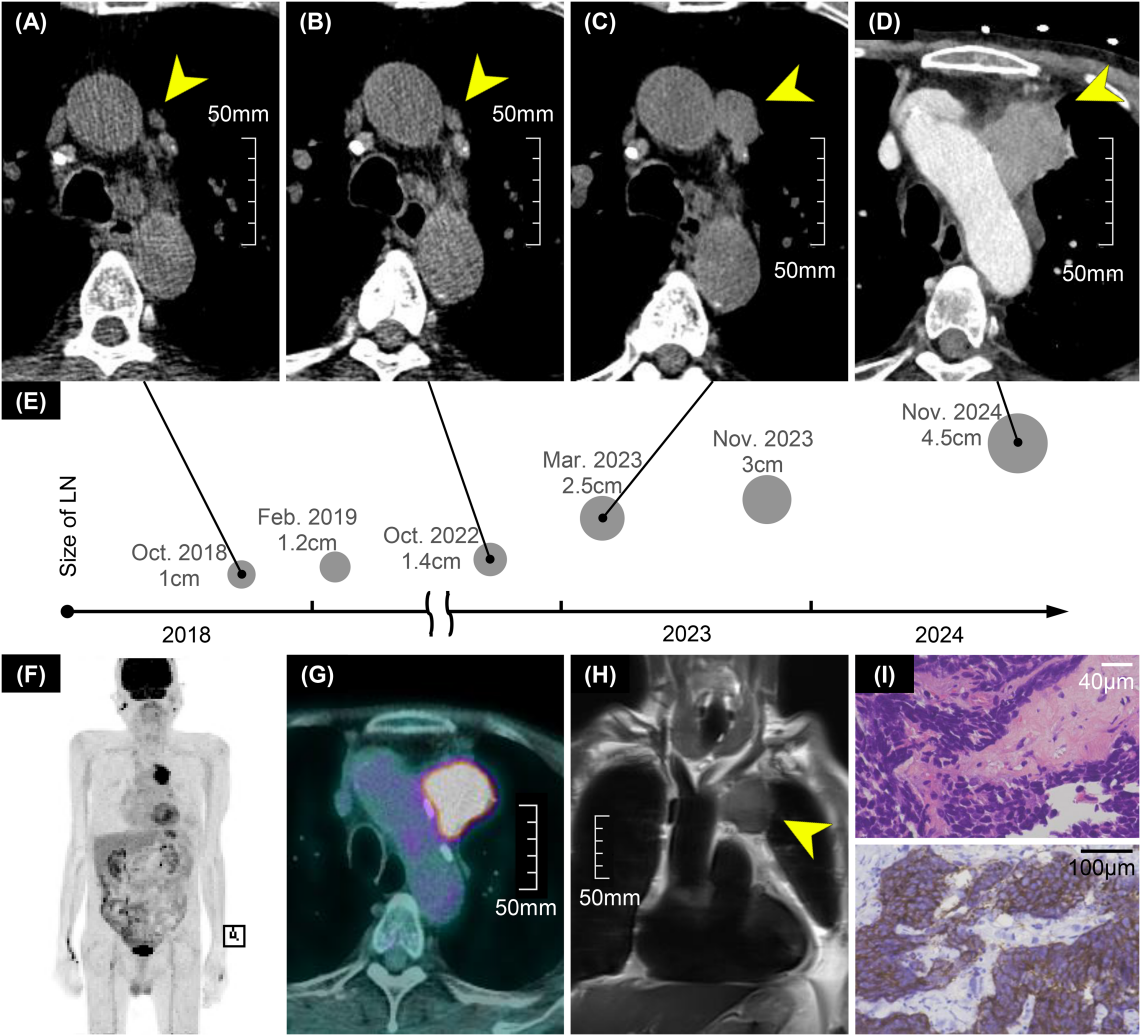
(A–D) Serial chest CT scans showing progressive enlargement of the para‐aortic lymph node (yellow arrowhead). (A) In October 2018, the node measured 1.0 cm in its long axis. (B) By October 2022, it had increased to 1.4 cm. (C) In March 2023, it had enlarged to 2.5 cm. (D) In November 2024, a contrast‐enhanced CT revealed further growth to 4.5 cm. (E) A timeline diagram illustrates the progression of lymph node enlargement. (F, G) FDG PET/CT demonstrated increased metabolic activity in the anterior mediastinum. (H) Chest MRI showed a mass with high T2 signal intensity, heterogeneous enhancement, and diffusion restriction (yellow arrowhead). (I) Haematoxylin and eosin staining revealed minimal cytoplasm, indistinct nucleoli, nuclear moulding, and smudging. Immunohistochemistry showed synaptophysin positivity (original magnification ×400).

Subsequent imaging, including contrast‐enhanced CT (Figure 1D), 2‐deoxy‐2‐[18F]‐fluorodeoxyglucose positron emission tomography/CT (Figure 1F,G), and magnetic resonance imaging (MRI) (Figure 1H), showed no evidence of distant metastasis. Incidental FDG avidity was observed in the parotid glands, but further neck MRI revealed these regions to be benign tumours such as Warthin's tumour. A percutaneous transthoracic needle biopsy of the anterior mediastinal mass confirmed the diagnosis of mediastinal small cell carcinoma (MSCC) (Figure 1I). Although concurrent chemoradiotherapy was planned, the patient declined further treatment.

The median survival of patients with untreated small cell lung cancer is approximately 3 months [1]. In contrast, we present a case of MSCC with extraordinarily slow progression, documented by long‐term CT follow‐up. In patients with chronic lung disease, lymphadenopathy—although frequently observed—should be evaluated with the same level of scrutiny as parenchymal lung lesions [2]. Close attention to mediastinal findings is essential to avoid delayed diagnosis of MSCC.

## Author Contributions

S.I. Ahn: investigation, data curation, writing – original draft, writing – review and editing, visualization. M.J. Chung: resources, investigation, validation. K.J. Chae: investigation, validation. J.S. Jeong: conceptualization, methodology, writing – review and editing, supervision, project administration, funding acquisition. Y.C. Lee: conceptualization, methodology, resources, writing – review and editing, supervision, project administration, funding acquisition.

## Funding

This work was supported by the National Research Foundation of Korea (NRF), funded by the Korean government (MSIT) (No. RS‐202400356349). Funding was also received from Korea Health Technology R&D Project through Korea Health Industry Development Institute (KHIDI), funded by the Ministry of Health & Welfare, Republic of Korea (No. RS‐2024‐00440408). Lastly, this paper was also supported by the Biomedical Research Institute fund at Jeonbuk National University Hospital.

## Ethics Statement

The study protocol was approved by the Institutional Review Board of Jeonbuk National University Hospital (No. 2024–02‐014). The authors declare that this study was conducted in accordance with the principles of the Declaration of Helsinki.

## Consent

The authors declare that written informed consent was obtained for the publication of this manuscript and accompanying images and attest that the form used to obtain consent from the patient complies with the Journal requirements as outlined in the author guidelines. All imaging data (CT, MRI, PET/CT) included in this report have been anonymized to protect the patient's identity. No personal identifiers, such as names, dates of birth, or facial features, are included.

## Conflicts of Interest

The authors declare no conflicts of interest.

## Data Availability

Data sharing not applicable to this article as no datasets were generated or analysed during the current study.
